# Intramural hematoma of colon: Case report of 2 cases

**DOI:** 10.1097/MD.0000000000019404

**Published:** 2020-03-06

**Authors:** Jing Wang, Xiaoyan Sun, Hongkun Shi, Dianbo Cao

**Affiliations:** Department of Radiology, First Hospital of Jilin University, Changchun, Jilin Province, China.

**Keywords:** aspirin, cancer, clopidogrel, colon, intramural hematoma

## Abstract

**Introduction:**

Colonic intramural hematomas are rarely encountered clinical entity. Colonic intramural hematomas are frequently associated with blunt trauma, and they could occur spontaneously in patients under anticoagulant therapy or with bleeding diathesis. There were few reports on synchronous colon cancer and intramural hematoma. Intramural hematomas of gastrointestinal tract in those patients undergoing anticoagulation treatment often occurred at the esophagus, duodenum, and small intestine, while colon was rarely affected site. Clinical symptoms of colonic intramural hematomas may include abdominal pain, lower gastrointestinal bleeding, and occasionally bowel obstruction.

**Patient concerns:**

We herein report 2 cases of colonic intramural hematomas. Case 1 presented with abdominal pain and decreased defecation. Colonoscopy and contrast-enhanced computed tomography (CT) revealed intramural hematoma proximal to the neoplasm at ascending colon. Case 2 was a patient under regular anticoagulation therapy after coronary arterial stent implantation. His chief complaints were intermittent abdominal pain and distension. Colonoscopy and contrast-enhanced CT demonstrated intramural hematoma at sigmoid colon.

**Diagnosis:**

Case 1 was diagnosed synchronous colonic intramural hematoma and colon cancer at ascending colon via surgery. Case 2 was diagnosed intramural hematoma of sigmoid colon through colonoscopy and follow-up CT.

**Interventions:**

Case 1 underwent right hemicolectomy. Case 2 received conservative treatment including anticoagulation discontinuation, total parenteral nutrition, and intravenous hydration.

**Outcomes:**

They both had a good recovery.

**Conclusion:**

Colonoscopy and CT are useful in diagnosing colonic intramural hematoma. The optimal treatment should be individualized according to different etiologies causing hematoma.

## 1 Introduction

Colonic intramural hematomas are rare but important clinical entities because of their inclination to cause hemorrhage. The etiologies may include abdominal trauma, anticoagulant therapy, or bleeding diathesis such as hemophilia and leukemia.^[1,2]^ Rarely did it occur as an iatrogenic consequence, or a rare complication of vaginal delivery.^[3]^ Few cases of synchronous colon cancer and intramural hematoma were reported. There are limited data on prevalence or treatment outcome of colonic intramural hematomas. We herein report 2 cases of colonic intramural hematomas with different pathogeneses, different treatment modalities, and both had a good recovery in the end.

## 2 Case report

### 2.1 Case 1

**Figure 1:**
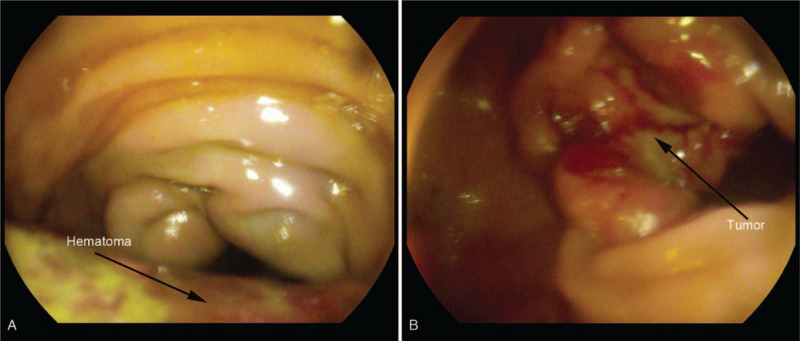
(A) Colonoscopy disclosed a large voluminous mass covered with violet and bluish mucosa suggestive of submucosal hemorrhage located at ascending colon. (B) A half circumferential ulcerative lesion with dirty surface on colonoscopic image was consistent with adenocarcinoma.

**Figure 2:**
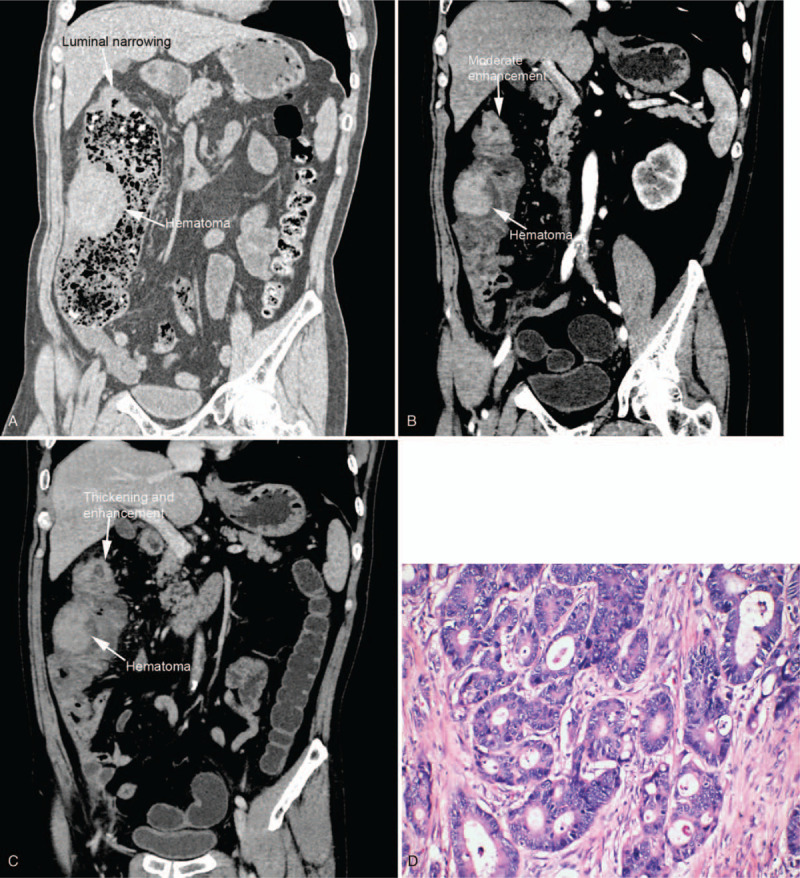
(A) Nonenhanced abdominal CT revealed thickening of colonic wall and luminal narrowing at hepatic flexure, and a hyperdense mass located on the right wall of ascending colon. (B and C) Arterial and venous phase of contrast enhanced CT showed unenhanced submucosal mass indicative of hematoma, while the thickened colonic wall at hepatic flexure enhanced moderately. (D) Postoperative histopathological findings were consistent with moderately differentiated adenocarcinoma (HE staining 20×). CT = computed tomography.

A 66-year-old man visited the emergency room complaining of abdominal pain and decreased defecation. His past medical history was unremarkable. Physical examination on admission showed mild tenderness on right abdomen. Serum tumor markers including carcinoembryonic antigen and carbohydrate antigen 72-4were slightly elevated to about twice the normal limits. Other laboratory data including blood cell counts, biochemical tests, and coagulation test were all within normal limits. Colonoscopy disclosed a large voluminous mass covered with violet and bluish mucosa suggestive of submucosal hemorrhage, 85 cm from the anal verge (Fig. 1A), and a half circumferential ulcerative lesion with dirty coating, 80 cm from the anal verge (Fig. 1B). Biopsy specimens of ulcerative lesion revealed adenocarcinoma. An abdominal computed tomography (CT) demonstrated a hyperdense mass locating at ascending colon (Fig. 2A), adjacent to the colon cancer at the hepatic flexure of colon. On contrast-enhanced CT done at the following day, the hyperdense mass showed no enhancement (Fig. 2 B and C). It was diagnosed colonic wall hematoma and colon cancer. The patient underwent right hemicolectomy. The histopathological examination revealed moderately differentiated adenocarcinoma with T3N1b staging (Fig. 2D). The postoperative recovery was uneventful.

### 2.2 Case 2

**Figure 3:**
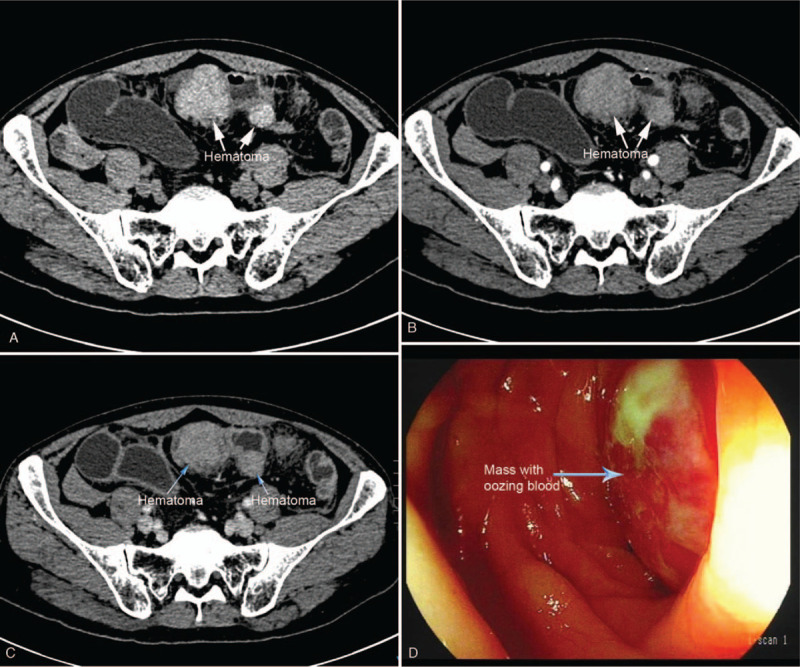
(A–C) Abdominal CT revealed multiple hyperdense masses in the sigmoid colon without enhancement after contrast administration. (D) Colonoscopy showed a submucosal mass with intact mucosa and oozing blood on its surface. CT = computed tomography.

**Figure 4:**
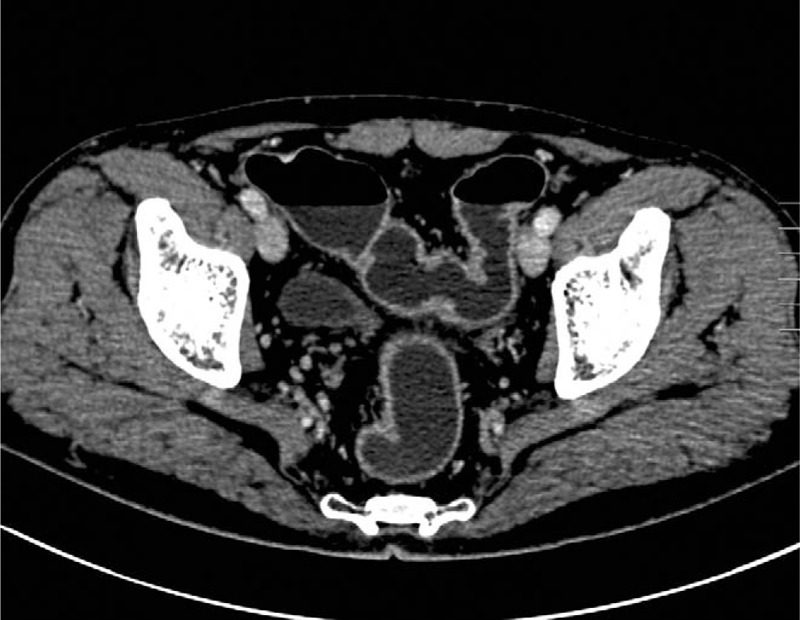
Abdominal CT image showed that the colonic masses resolved completely at the 1-month follow-up. CT = computed tomography.

A 57-year-old man was admitted to our hospital with complaint of intermittent abdominal pain and distension. He underwent coronary stent angioplasty 1 month ago and had been taking aspirin and clopidogrel since then. He denied recent trauma history. Coagulation test showed an elevated fibrinogen of 4.58 g/L (2.0–4.0 g/L). Abdominal CT scan revealed multiple hyperdense masses obstructing the sigmoid colon (Fig. 3A) without significant enhancement after contrast administration (Fig. 3 B and C). Colonoscopy showed a submucosal mass with intact mucosa and oozing blood on its surface which almost totally occluded the lumen (Fig. 3D). He was diagnosed intramural hematoma of sigmoid colon. Given the abnormality of coagulation function, anticoagulation drugs were discontinued and conservative treatment including total parenteral nutrition and intravenous hydration was initiated in time, without blood transfusion. Follow-up abdominal CT 1 month later indicated that the colonic masses completely resolved (Fig. 4). Then he began to take cilostazol and clopidogrel instead of aspirin. There was no recurrence of hemorrhage during 1 year of follow-up.

## 3 Discussion

Colonic intramural hematomas are rare. They could occur at any segment of the colon.^[2]^ The rectum and sigmoid colon were relatively commonly involved sites.^[4]^ The etiologies may include abdominal trauma, anticoagulant therapy, or bleeding diathesis such as hemophilia and leukemia.^[1,2]^ It is extremely rare for them to occur as an iatrogenic injury such as double-balloon enteroscopy,^[5]^ stapled hemorrhoidopexy,^[6]^ polypectomy,^[7]^ or occurred as a rare complication of vaginal delivery.^[3]^ Besides, submucosal hematoma of the colon was once reported to happen on an immunoglobulin light-chain amyloidosis patient who presented with hematochezia. The possible mechanisms in immunoglobulin light-chain amyloidosis include vascular fragility and acquired factor X deficiency.^[8]^ There were few reports on synchronous colon cancer and submucosal hematoma.^[9]^ In our case 1, it demonstrated intramural hematoma proximal to colon cancer. The patient did not have any trauma, anticoagulant, or other related history. The exact pathogenesis was perplexing us. As shown on CT, there was typical ileus secondary to colonic neoplasm, so intraluminal pressure of proximal colon increased suddenly which was triggered by strong intestinal peristalsis. The hematoma may be associated with the broken continuity or rupture of terminal arteries on colonic wall under these circumstances. Intramural hematomas in patients under anticoagulation therapy often occurred at the esophagus, duodenum, and small intestine, and colon is rarely involved.^[10–12]^ It was reported that the incidence of small intestinal submucosal hematoma in patients with anticoagulant therapy was about 1 per 25,000 each year, and that of colonic submucosal hematomas was even a lower ratio.^[11]^ For our case 2, he was receiving anticoagulant therapy, and the coagulation test was abnormal, so we can confidentially ascribe to it. Meanwhile, the natural evolution of hematomas after anticoagulation discontinuation further confirmed our initial judgment.

The severity of clinical symptoms varies, which include abdominal pain, bowel obstruction, lower gastrointestinal bleeding, hemorrhagic shock, etc. Physical examination may reveal localized or diffused abdominal tenderness and peritoneal irritation. Most cases of colonic intramural hematomas could be diagnosed by endoscopy and CT. Colonoscopy could reveal a localized, submucosal mass with different manifestations of mucosa. For our patient secondary to colon cancer, the mucosa was violet and bluish suggestive of broken continuity or rupture of terminal arteries, and colon cancer was visualized simultaneously. For the patient under anticoagulant therapy, it showed a reddish intact mucosa with oozing blood on its surface. In some stable patients with unclear diagnosis, biopsy and pathological examination may help differentiate a hematoma from a neoplasm, but it carries a high risk of bleeding. CT is very useful in diagnosing colonic intramural hematomas whose sensitivity is nearly 100%.^[11]^ CT can not only define the areas of hemorrhage, but also point out possible complications and its cause. Intramural hematomas may present as eccentric or circumferential thickening of the bowel wall,^[13]^ luminal narrowing, intramural hyperdensity, and bowel obstruction.^[11]^ Echoendoscopy was also reported to be used in diagnosing intramural hematoma at accessible location of the lesion. Echoendoscopy is useful in determining the thickness of the wall and infiltration, also in evaluating echostructures of the lesion.^[14]^

The optimal management depends on different etiologies and the patient's general condition. In stable patients especially with bleeding diathesis, conservative treatment is usually the preferred option.^[2,4,5,15]^ As our case 2, the intramural hematoma resolved completely after a short interval of conservative treatment. Although in selected cases, surgical drainage can be helpful,^[2]^ and in some serious situations, emergency surgery may be necessary.^[1,12]^ The indications for laparotomy include hemodynamic instability, generalized peritonitis, serious compression or obstruction, and the presence of contrast agent extravasation on dynamic contrast enhanced CT scan, a clue of active bleeding.^[3,11]^ Intramural hematomas resulting from blunt colonic injuries often require timely surgical intervention, because the patients are in danger of serious complications such as sepsis and abscess.^[13]^ Prompt surgery is crucial for preventing complications and improving outcomes in patients with blunt colonic injuries. For patients secondary to other pathologies, surgery is necessary to dislodge the cause. As described in case 1, right hemicolectomy removed the neoplasm and intramural hematoma simultaneously and resulted in a good recovery.

Two cases of colonic intramural hematomas with different pathogeneses were described. Their diagnoses are established via abdominal CT investigation and colonoscopy before the treatment. Surgery is necessary to dislodge some causes such as neoplasm, while conservative treatment is reserved for those stable patients with bleeding diathesis.

## Author contributions

**Conceptualization:** Jing Wang, Dianbo Cao.

**Data curation:** Xiaoyan Sun.

**Methodology:** Hongkun Shi.

**Resources:** Xiaoyan Sun, Hongkun Shi.

**Writing – original draft:** Jing Wang.

**Writing – review & editing:** Jing Wang, Dianbo Cao.

Dianbo Cao orcid: 0000-0002-4456-9640.
